# Urine ethanol concentration and alcohol hangover severity

**DOI:** 10.1007/s00213-016-4437-0

**Published:** 2016-09-28

**Authors:** Aurora Van de Loo, Marlou Mackus, Gerdien Korte-Bouws, Karel Brookhuis, Johan Garssen, Joris Verster

**Affiliations:** 1 Division of Pharmacology, Utrecht University, Universiteitsweg 99, 3584CG, Utrecht, The Netherlands; 2 Faculty of Behavioral and Social Sciences, Groningen University, Groningen, The Netherlands; 3 Nutricia Research, Utrecht, The Netherlands; 4 Centre for Human Psychopharmacology, Swinburne University, Melbourne, Australia

**Keywords:** Ethanol, Alcohol, Hangover, Severity

## Abstract

**Background:**

The aim of this study was to examine the relationship between urine ethanol concentration and alcohol hangover severity.

**Methods:**

*N* = 36 healthy social drinkers participated in a naturalistic study, comprising a hangover day and a control day. *N* = 18 of them have regular hangovers (the hangover group), while the other *N* = 18 claim to be hangover immune (hangover-immune group). On each test day at 9.30 am, urine samples were collected. Participants rated their overall hangover severity on a scale from 0 (absent) to 10 (extreme), as well as 18 individual hangover symptoms.

**Results:**

Urine ethanol concentration was significantly higher on the hangover day when compared to the control day (*p* = 0.006). On the hangover day, urine ethanol concentration was significantly lower in the hangover-immune group when compared to the hangover group (*p* = 0.027). In the hangover-immune group, none of the correlations of urine ethanol concentration with individual hangover symptoms was significant. In contrast, in the hangover group, significant correlations were found with a variety of hangover symptoms, including nausea, concentration problems, sleepiness, weakness, apathy, sweating, stomach pain, thirst, heart racing, anxiety, and sleep problems.

**Conclusion:**

Urine ethanol levels are significantly associated with the presence and severity of several hangover symptoms.

## Introduction

Alcohol hangover is often described as the combination of symptoms experienced the day after an evening of heavy alcohol consumption (Verster et al. 2010). Frequently reported symptoms include being tired, thirsty, drowsiness, sleepiness, headache, dry mouth, nausea, weakness, concentrations problems, and reduced alertness (Penning et al. 2012). A factor analysis to categorize 49 hangover-related symptoms revealed that the two most important factors were related to drowsiness and impaired cognitive functioning (Penning et al. 2012).

The relationship between the amount of consumed alcohol and the presence and severity of hangover symptoms is not straightforward. Although at a group level, it is correct that hangovers are usually more severe if more alcohol has been consumed; this is not always true for individual drinkers (Penning et al. 2010). Moreover, after consuming the same amount of alcohol, a drinker may experience a hangover on one occasion, but not on a comparable other drinking occasion. Thus, the presence and severity of hangover symptoms vary between drinkers and may also vary between drinking occasions. Only few studies examined this topic and reported inconclusive results. For example, Ylikahri et al. (1974) found no significant correlation between peak blood alcohol concentration and hangover severity in 23 healthy students after drinking alcohol (1.5 g/kg), whereas Kruisselbrink et al. (2006) did report a significant relationship between hangover severity and the administered dose of alcohol. To complicate matters, there is also a group of drinkers that claim not to experience hangovers, i.e., the hangover-immune group, despite consuming large quantities of alcohol. A review of both controlled experimental studies and surveys (Howland et al. 2008) concluded that this group of hangover-immune drinkers comprises about 25 % of social drinkers. In real life, however, peak BAC values can be much higher than those administered in experimental studies (Verster et al. 2015). Data from a large survey among Dutch students revealed that the majority of drinkers who claim to be hangover immune do not reach an estimated BAC of 0.08 % (Verster et al. 2013) and confirmed that most social drinkers who claim to be hangover immune simply do not drink sufficient quantities of alcohol to provoke a hangover per se. The data showed that with increasing estimated BACs, the percentage of drinkers claiming to be hangover immune reduces significantly. Nevertheless, a small percentage of heavy drinkers persists in claiming to be hangover immune, despite having peak BACs above 0.15 %.

The current study examined the relationship between breath and urine ethanol concentration and hangover severity in social drinkers, of which half reported a hangover and the other half claimed to be hangover immune after a heavy drinking session.

## Materials and methods

### Study design

This naturalistic study comprised a hangover day and a control day (no alcohol consumed the previous day). Data were collected on test days following real-life drinking sessions. No constraints are imposed upon participants’ behavior in this observational study, and the investigators were not present during the drinking session or on the control day. The University of Groningen Psychology Ethics Committee approved the study. Written informed consent was obtained before the start of the study.

### Participants

Healthy social drinkers, 18–30 years old, were recruited to participate in the study. They should report having occasions on which they consume at least five alcoholic beverages, at least three times per month. The advertisement made clear that we were recruiting two types of social drinkers: (1) those who have hangovers after an evening of alcohol consumption and (2) those that do not have hangovers after an evening of alcohol consumption. To verify whether participants consume sufficient amounts of alcohol to produce an alcohol hangover per se, their estimated peak breath alcohol concentration (BAC) on such occasions was computed. Estimated BAC should be above 0.08 % and was computed using the formula of Mathews and Miller (1979), taking into account the number of drinks consumed within a certain time frame, controlling for gender and body weight. Drug use was verified using urine drug tests on the test days, and participants who scored positive were excluded from participation. Participants were allocated to one of two groups: (1) *N* = 18 participants who report experiencing hangovers after reaching this estimated BAC and (2) *N* = 18 participants who reported that they never experiencing alcohol hangovers, despite regularly consuming alcohol corresponding to estimated BACs higher than 0.08 %.

### Procedures

Participants consumed alcohol (or not) at their own pace, quantity, and in a setting of their own choice. Test days were postponed if participants chose not to consume alcohol. Participants were asked not to consume alcohol at least 24 h before the control test day, not to use recreational drugs, and not to consume any caffeinated beverages on test days.

On both the hangover day and a control day, a urine sample was collected at 9.30 am, and several subjective assessments were made. Urine was tested (InstantView) for the presence of amphetamines (including MDMA), barbiturates, cannabinoids, benzodiazepines, cocaine, and opiates, and the remaining urine was stored for ethanol determination.

Participants completed a questionnaire regarding last evening’s drinking behavior (including start and stop time of alcohol consumption and the number and type of drinks consumed) to enable calculation of the estimated peak BAC for each participant. Overall hangover severity and the severity of 18 individual hangover symptoms were assessed, including headache, nausea, concentration problems, regret, sleepiness, heart beating, vomiting, tired, shaking, clumsy, weakness, dizziness, apathy, sweating, stomach pain, confusion, light sensitivity, thirst, heart racing, anxiety, depression, reduced appetite, and sleep problems. The presence and severity of overall hangover severity and each symptom individually were rated using 10-point scales, ranging from absent (0) to extreme (10).

### Urine collection, handling, and analysis

On each test day, a urine sample was collected at 09.30 am. Any turbid urine samples were centrifuged at 3000 rpm for 15 min at room temperature. The urine was stored in three 3-mL cryovials, at a temperature of −20 °C. Urine ethanol concentration was determined using headspace gas chromatography with flame ionization detection. The separation was performed on a Porapak Q packed column. Samples were spiked with 1-propanol as an internal standard (IS). Together with the headspace syringe, the samples were heated at 80 °C for 20 min. The gas phase in the vial was manually injected into the column of the headspace chromatograph. The temperature of the column oven was set at 140 °C for 9 min. After the elution of the IS, the temperature was increased to 200 °C for 5 min to remove any late eluting sample compounds from the column. For each standard curve sample, peak ratio of ethanol to the IS was calculated and plotted against the ethanol concentration. Results from urine samples from participants were plotted in the calibration curve, calculating ethanol concentrations using the corresponding formula.

### Statistical analyses

Statistical analyses were performed with SPSS, version 23. Urine ethanol concentration of the hangover and control day were compared using the Mann-Whitney U test. Delta scores (hangover—control day) were computed for each variable. Delta ethanol concentration was correlated (nonparametric, Spearman’s rho) with overall hangover severity and severity scores for the individual hangover symptoms. The analyses were conducted for all participants together (*N* = 36) and separate for the hangover group (*N* = 18) and the hangover-immune group (*N* = 18).

## Results

Participants of the hangover-immune group (*N* = 18) and hangover group (*N* = 18) respectively did not differ significantly in mean ± SD age (20.8 ± 2.0 versus 21.4 ± 1.6 years old), height (1.79 ± 0.1 versus 1.76 ± 0.1 m), and weight (71.1 ± 10.2 versus 67.2 ± 11.5 kg). Regarding alcohol consumption, the hangover-immune and hangover group did not differ on the total number of alcoholic drinks consumed (10.7 ± 4.7 versus 12.5 ± 7.3 drinks) and the estimated BAC that was achieved (0.17 ± 0.07 versus 0.19 ± 0.09 %). As expected, overall hangover severity was significantly higher in the hangover group when compared to the hangover-immune group (5.6 ± 2.4 versus 0.7 ± 1.4, *p* = 0.006).

Table 1 gives an overview of the urine ethanol concentrations, as determined on the hangover and control day.

**Table 1:** Urine ethanol determinations

Hangover group urine ethanol (mg/L)			Hangover-immune group urine ethanol (mg/L)		
Subject	Control day	Hangover day	Subject	Control day	Hangover day
S101	11.91	48.36	S201	1.23	6.28
S102	0.52	3.58	S202	1.94	6.20
S103	0	476.03	S203	1.35	0.60
S104	1.27	338.26	S204	2.03	2.65
S105	0.45	366.12	S205	0	2.44
S106	0.18	2.34	S206	1.25	42.32
S107	1.66	1.24	S207	3.93	1.47
S108	2.18	438.79	S208	0	436.69
S109	7.98	0.87	S209	1.96	3.22
S110	1.94	800.18	S210	2.17	1.99
S111	1.93	2.63	S211	0.58	2.51
S112	0.25	307.92	S212	0.85	26.44
S113	0.32	1.01	S213	0.24	0
S114	0.21	0.93	S214	0.25	9.34
S115	11.27	1.59	S215	0.4	0.60
S116	5.04	431.92	S216	0.46	3.35
S117	1.93	77.21	S217	0.5	1.07
S118	0.37	35.84	S218	1.08	61.66
*Mean*	*2.75*	185.27*	*Mean*	*1.12*	*33.82*
*SD*	*3.78*	*240.23*	*SD*	*1*	*101.93*
*Range*	*0–11.91*	*0.87–800.18*	*Range*	*0–3.93*	*0–436.69*

Mean, standard deviation (SD), and range are presented. Significant differences (*p* < 0.05) between the hangover group and hangover-immune group are indicated by an asterisk

On the hangover day, residual ethanol was present in 31 of 36 corresponding urine samples (86.1 %). Overall (*N* = 36), the urine ethanol concentration (*p* = 0.002) was significantly higher on the hangover day when compared to control day. For the hangover group, on the hangover day, a significantly higher urine ethanol concentration (*p* = 0.006) was found when compared to the control day. For the hangover-immune group, no significant differences were observed for urine ethanol levels between the hangover and control day. Urine ethanol concentration on the hangover day was significantly higher in the hangover group when compared to the hangover-immune group (*p* = 0.027). No differences between the two groups were observed on the control day.

The 1-item hangover severity score did not significantly correlate with urine ethanol concentration. Regarding individual hangover symptoms, overall (*N* = 36), a significant correlation was found with nausea (*r* = 0.386, *p* = 0.020), headache (*r* = 0.392, *p* = 0.018), and sleep problems (*r* = 0.364, *p* = 0.029). When analyzing the data for the two groups separately, a clear distinction became evident (see Table 2).

**Table 2:** Correlations between urine ethanol concentration and hangover symptom severity

	Hangover group	Hangover-immune group
1-item overall hangover score	0.571**	0.307
Sleepiness	0.646**	0.094
Sweating	0.640**	0.172
Concentration problems	0.633**	0.102
Nausea	0.602**	0.310
Thirst	0.599**	0.149
Sleep problems	0.552*	0.255
Heart racing	0.550*	−0.340
Dizziness	0.534*	0.150
Confusion	0.520*	0.016
Shaking	0.517*	0.017
Headache	0.513*	0.255
Regret	0.512*	0.017
Weakness	0.492*	0.236
Clumsy	0.463	0.118
Stomach pain	0.422	0.146
Heart beating	0.422	−0.183
Anxiety	0.411	−0.341
Depression	0.400	0.034
Reduced appetite	0.379	−0.180
Light sensitivity	0.299	0.371
Vomiting	0.286	−0.141
Tired	0.165	0.299
Apathy	0.081	−0.108

Significant correlations are indicated by *(*p* < 0.05) or **(*p* < 0.01)

In the hangover-immune group, none of the correlations of ethanol concentration with individual hangover symptoms was significant. In contrast, in the hangover group, significant correlations were found between urine ethanol concentration and nausea (*r* = 0.602, *p* = 0.008), headache (*r* = 0.513, *p* = 0.030), concentration problems (*r* = 0.633, *p* = 0.005), regret (*r* = 0.512, p = 0.030), sleepiness (*r* = 0.646, *p* = 0.004), shaking (*r* = 0.517, *p* = 0.028), weakness (*r* = 0.492, *p* = 0.038), dizziness (*r* = 0.534, *p* = 0.022), sweating (*r* = 0.640, p = 0.004), confusion (*r* = 0.520, *p* = 0.027), thirst (*r* = 0.599, *p* = 0.009), heart racing (*r* = 0.550, *p* = 0.018), and sleep problems (*r* = 0.552, *p* = 0.017).

## Discussion

Next day urine ethanol concentration was significantly higher on hangover day compared to control day. Also, urine ethanol concentration was significantly higher in the hangover group when compared to the hangover-immune group. In the hangover-immune group, none of the correlations of urine ethanol concentration with individual hangover symptoms was significant. In contrast, in the hangover group, significant correlations were found with a variety of hangover symptoms, including nausea, headache, concentration problems, regret, sleepiness, shaking, weakness, dizziness, sweating, confusion, thirst, heart racing, and sleep problems.

Thus, among participants who experienced a hangover, a clear significant relationship was observed between urine ethanol concentration and a variety of hangover symptoms. This association was not seen in participants claiming to be hangover free. No differences in demographics or alcohol consumption were observed between the hangover group and the hangover-immune group that can explain our findings. For example, total alcohol consumed and estimated peak BAC did not significantly differ between the hangover group and the hangover-immune group.

It can be hypothesized that the difference between the hangover group and hangover-immune group regarding urine ethanol concentrations and experiencing hangover symptoms is related to variations in alcohol metabolism. Administration of ethanol causes the production of acetaldehyde as an intermediate metabolite. Aldehyde dehydrogenase (ALDH) further metabolizes acetaldehyde to acetate. Perhaps, fast metabolizers of ethanol and acetaldehyde do not reach peak BAC levels that would be expected when using common formulas such as those by Matthews and Miller (1979). Rapid elimination of alcohol could be an explanation why fast metabolizers may claim to be hangover immune, despite consuming the same amount of alcohol as slow metabolizers. Further studies are needed to examine the role of ethanol metabolism in the pathology of alcohol hangover. In such studies, alcohol should be administered to achieve a target BAC in participants with a hangover versus hangover-immune subjects. Then, the realized peak BAC and the rate of ethanol elimination can be directly compared. If such a study confirms a lower peak BAC and faster ethanol elimination in hangover-immune drinkers, this would support the idea that fast metabolizers may be hangover immune because they do not reach high enough BAC levels to provoke a hangover (Verster et al. 2013).

Alternatively, not ethanol itself but its metabolites may be related to experiencing hangover symptoms and have an impact on their severity. For example, several studies have suggested that acetaldehyde concentration may also be an important factor determining the presence and severity of alcohol hangover (Eriksson 1983; Tsukamoto et al. 1991; Ylikahri et al. 1974). During analyses of our urine samples for ethanol content, two unidentified peaks appeared in each chromatogram at a retention time of 2.348 and 6.593. Based on literature, a trial run was performed on the congeners 2-butanol, iso-butanol, 2-methyl-1-butanol, 3-methyl-1-butanol, and 1-propanol, and metabolites of ethanol acetaldehyde, and acetone. The trial runs were repeated three times, using different gradients. The first peak was identified to be acetaldehyde (RT = 2.348), the second as acetone (RT = 6.593). Future analyses should investigate the potential role of acetaldehyde in the pathology of alcohol hangover.

Although the observed associations have considerable strength, some limitations of the current study should be addressed. Limitations that are common to a naturalistic study design also apply to the current investigation. For example, alcohol consumption, sleep time, and participant behavior in general were not controlled by the investigators. Although all urine samples were collected around 9.30 am, the time between stopping alcohol consumption and sample collection varied between participants. Further, although (alcoholic and non-alcoholic) beverage consumption was recorded, it is not known to what extent participants consumed water during the night, nor was it recorded whether or not they urinated during the night or early morning. Voiding urine during this period of time may have caused excretion of ethanol. It is unclear to what extent these variations may have had an impact on the study outcome. On the other hand, at a group level, variables related to alcohol consumption, sleep, and demographics do not differ significantly between the hangover group and the hangover-immune group, suggesting that their impact on the study outcome is limited.

Taken together, in the hangover group ethanol levels showed to be significantly associated with the presence and severity of several hangover symptoms. In contrast, in the hangover-immune group ethanol concentration on the hangover day did not differ significantly from the control day, and these individuals did not report experiencing typical hangover symptoms. This observation suggests that hangover-immune individuals may be fast metabolizers of alcohol, that do not reach the peak BAC required to provoke an alcohol hangover. More research into the association between hangover severity and ethanol metabolism is therefore warranted.
